# Donor-Acceptor-Type Copolymers Based on 3,4-Propylenedioxy-thiophene and 5,6-Difluorobenzotriazole: Synthesis and Electrochromic Properties

**DOI:** 10.3390/polym10040427

**Published:** 2018-04-11

**Authors:** Fanda Feng, Lingqian Kong, Hongmei Du, Jinsheng Zhao, Junhong Zhang

**Affiliations:** 1 Shandong Key Laboratory of Chemical Energy Storage and Novel Cell Technology, Liaocheng University, Liaocheng 252059, China; 18863500270@163.com (F.F.); duhongmei@lcu.edu.cn (H.D.); 2 Dongchang College, Liaocheng University, Liaocheng 252059, China; lingqiankong@126.com

**Keywords:** D-A-type polymers, 5,6-difluorobenzotriazole, propylenedioxythiophene, electrochromic property

## Abstract

Three solution-processable D-A-type conjugated polymers **P1**, **P2** and **P3** were successfully synthesized via the Pd-catalyzed Stille cross-coupling copolymerization approach, with 6,8-Dibromo-3,3-bis-decyl-3,4-dihydro-2*H*-thieno[3,4-*b*][1,4] dioxepine (M1) and 2,5-Bis(trimethylstannanyl)thiophene (M3) as the donor units and 4,7-Dibromo-5,6-difluoro-2-(2-hexyl-decyl)-2*H*-benzotriazole (M2) as the acceptor unit, wherein the feed ratio of the three units was 1:3:4 (M1:M2:M3, the same below), 1:1:2 and 3:1:4 for **P1**, **P2**, and **P3**, respectively. The results obtained by our test showed that the feed ratio between the D and A units had a significant effect on both the electrochemical and the spectroelectrochemical properties of the three polymers. The copolymers exhibited a gradually deepening red color in neutral state with the increase of M1 content and then turned to a transmissive grey color in the oxidation state. Also, three copolymers showed good performance in electrochromic parameters, which mainly consists of optical contrast, response time, and coloration efficiency. In general, the excellent electrochromic performances of the copolymers make them outstanding candidates for electrochromic material applications.

## 1. Introduction

Since the first conductive polymer-doped polyacetylene was discovered in the 1970s [1,2], research in conductive polymers has not stopped and mainly includes thiophene [3,4], furan [5], pyrrole [6], and their derivatives. Conductive polymers have wide applications in nanocomposites [7], thin films [8], solar cells [9], light emitting diodes [10], and electrochromic devices due to the discovery of novel polymers with excellent properties in optical and electrochromic aspects [11,12,13]. The electrochromic phenomenon is mainly manifested as a reversible optical change in the doping-dedoping process. Generally, electrochromic conjugated polymers (Ecps) will change from, for example, red, green, and other different neutral state colors to a slight gray color of the oxidation state when different voltages are applied [14,15,16]. 

Band gap is an important factor affecting the optical properties of Ecps, since the oxidation and reduction process of low band gap polymers can be achieved at considerably low potentials. Research on low band gap Ecps has attracted the attention of many scientists. To the best of our knowledge, there are four main approaches used to build soluble, low band gap polymers. The first method is focused on the synthesis of poly(thienylenevinylene) polymers and their analogues [17,18]. The second method is related to the increase of the quinone character of the polymer backbone [19], and the third approach is characterized by the purposeful control of the region regularity of polythiophenes [20]. The fourth and most commonly used approach is called the donor-acceptor (D-A) method, which has the best synergistic effect between the alternately existing donor and acceptor units on the polymer backbone [21,22] and is generally considered the most effective way to reduce the band gaps of polymers. 

As one of the most popular monomers, 3,4-ethylenedioxythiophene (EDOT) is highly studied by scientists due to its prominent merits, which include a high electron donating ability caused by the overlap between the lone pairs of electrons, oxygen atom, and thiophene ring, as well as the ability to form linear chains due to the blocking of the 3,4 positions of the thiophene ring [23]. The polymer of EDOT (PEDOT) can be simply prepared using the electrochemical or chemical oxidation method with many excellent properties including environmental stability, conductivity, and low bandgapS [24]. 3,4-propylene-dioxythiophene (ProDOT) has a structure similar to that of EDOT and exhibits superior properties in optical and electrochromic aspects [24,25,26]. Simultaneously, ProDOT monomer can be modified by various chemical strategies to optimize the solubility of its polymers, such as the introduction of new alkyl or alkoxy side chains. Besides, linear polymers can also be easily obtained through the polymerization of the monomers [26]. As a donor unit, 5,6-Difluorobenzotriazole (FBTA) offers many advantages in building conjugated D-A type polymers over simple benzotriazole-based polymers [27]. Two fluoro substitutions at positions five and six of the FBTA can reduce both the HOMO and LUMO values at the same time [27]. Meanwhile, the fluorine atom substitute can also lead to stronger intermolecular interactions of the synthetic polymer [27]. The introduction of the alkyl side chain at position 1 increases the solubility of the synthetic polymer, which makes the FBTA a good candidate for the preparation of Ecps as the acceptor units [28]. A few of D-A type conjugated polymers have been designed and synthesized based on FBTA as the acceptor unit, and the corresponding donor units included benzodithiophene (BDT) [29], benzo[1,2-*b*:4,5-*b*′]dithiophene (BDTT) [30], BDTT with octylthio chains [30], 3-hexylthiophene [31], terthiophene and quaterthiophene [28]. The studies on the application of the above resulting copolymers are mainly focused on the field of bulk heterojunction (BHJ) solar cells and field effect transistors. Of these devices, the FBTA containing polymers showed excellent performance. However, the application of these materials as electrochromic materials has rarely been studied, and requires attention in further research works. As far as we know, the copolymers consisting of FBTA, ProDOT, and thiophene have never been reported and their electrochromic properties are worth pursuing. In addition, the bandgaps and performances of the copolymers could be finely tuned by the optimization of the feed ratios of the donor units to the acceptor units. 

In this paper, we selected 4,7-Dibromo-5,6-difluoro-2-(2-hexyl-decyl)-2*H*-benzotriazole (M2) as the acceptor unit, 2,5-Bis(trimethylstannanyl)thiophene (M3), and 6,8-Dibromo-3,3-bis-decyl-3,4-dihydro-2*H*-thieno[3,4-*b*][1,4] dioxepine (M1) as the donor units to construct D-A-type conjugated polymers. The feed ratio of the donor units to the acceptor unit was adjusted by varying the contents of the M1 and M2 monomers. Three random copolymers were synthesized via the Stille cross-coupling approach and then a series of detailed analyses, including cyclic voltammetry (CV), spectral electrochemistry, kinetics, and colorimetry, were carried out, respectively [32]. Based on the data obtained, we systematically discussed the influence of the different portions of the donor units (or acceptor units) on the electrochemical and electrochromic properties of the polymers. The results showed that the increase of M1 content is beneficial to the electrochromic properties of the polymers, while the M2 is just the opposite, which is the main contribution of the present work. 

## 2. Experimental

### 2.1. Materials

All reagents and solvents, unless otherwise noted, were used as received without further purification. The synthetic methods for 6,8-dibromo-3,3-didecyl-3,4-dihydro-2*H*-thieno[3,4-*b*][1,4]dioxepine (M1) and its related intermediates including 2,2-Bis-decyl-propane-1,3-diol (BDP) and 3,3-Didecyl-3,4-dihydro-2*H*-thieno[3,4-*b*][1,4]dioxepine (ProDOT-decyl_2_) were referred to the previous literature [32,33]. 4,7-Dibromo-5,6-difluoro-2-(2-hexyl-decyl)-2*H*-benzotriazole (M2) and 2,5-Bis(trimethylstannanyl)thiophene (M3) were bought from Derthon Optoelectronic Materials Science Technology Co., Ltd. (Shenzhen, China). Indium tin oxide (ITO)-coated glass, was purchased from Zhuhai Kaivo Optoelectronic Technology Co., Ltd. (Zhuhai, China), was cleaned with ethanol, acetone, and deionized water sequentially under ultrasonic conditions before use. Other reagents including diethyl malonate, toluene, sodium hydride, lithium aluminum hydride, 1-bromodecane, acetone, ethanol, 3,4-dimethoxythiophene, *p*-methylbenzenesulfonic acid, acetonitrile (ACN), acetone, methanol are all analytical reagent (AR) and were obtained from Aladdin Industrial Corporation (Shanghai, China). Tetrabutylammonium hexafluorophosphate (TBAPF_6_) was obtained from Tokyo Chemical Industry Co., Ltd. (TCI, Tokyo, Japan).

### 2.2. Characterizations

^1^H NMR and ^13^C NMR spectra were taken by using a Varian AMX 400 spectrometer (Varian Inc., Santa Clara, CA, USA) with tetramethylsilane (TMS) as the internal reference. UV-Vis-NIR spectra were recorded using a Varian Cary 5000 spectrophotometer (Varian Inc., Santa Clara, CA, USA). Scanning electron microscope (SEM) measurements were recorded by using a Hitachi SU-70 thermionic field emission SEM (Hitachi, Ltd., Tokyo, Japan). Cyclic voltammetry (CV) was carried out on a CHI760 electrochemical workstation (CHI Ltd, Shanghai, China) with an ITO glass electrode used as the working electrode, which was spray-coated with polymer films. A platinum wire was used as the counter electrode and an Ag wire as the reference electrode. The films of the polymer were sprayed onto the ITO glass by a spray gun with an active area of 0.9 cm × 3.0 cm. The supporting electrolyte was prepared by dissolving 0.2 M TBAPF_6_ in ACN and was used throughout all studies [32].

### 2.3. Synthesis Process

#### 2.3.1. Synthesis of ProDOT-decyl_2_

**Scheme 1:**
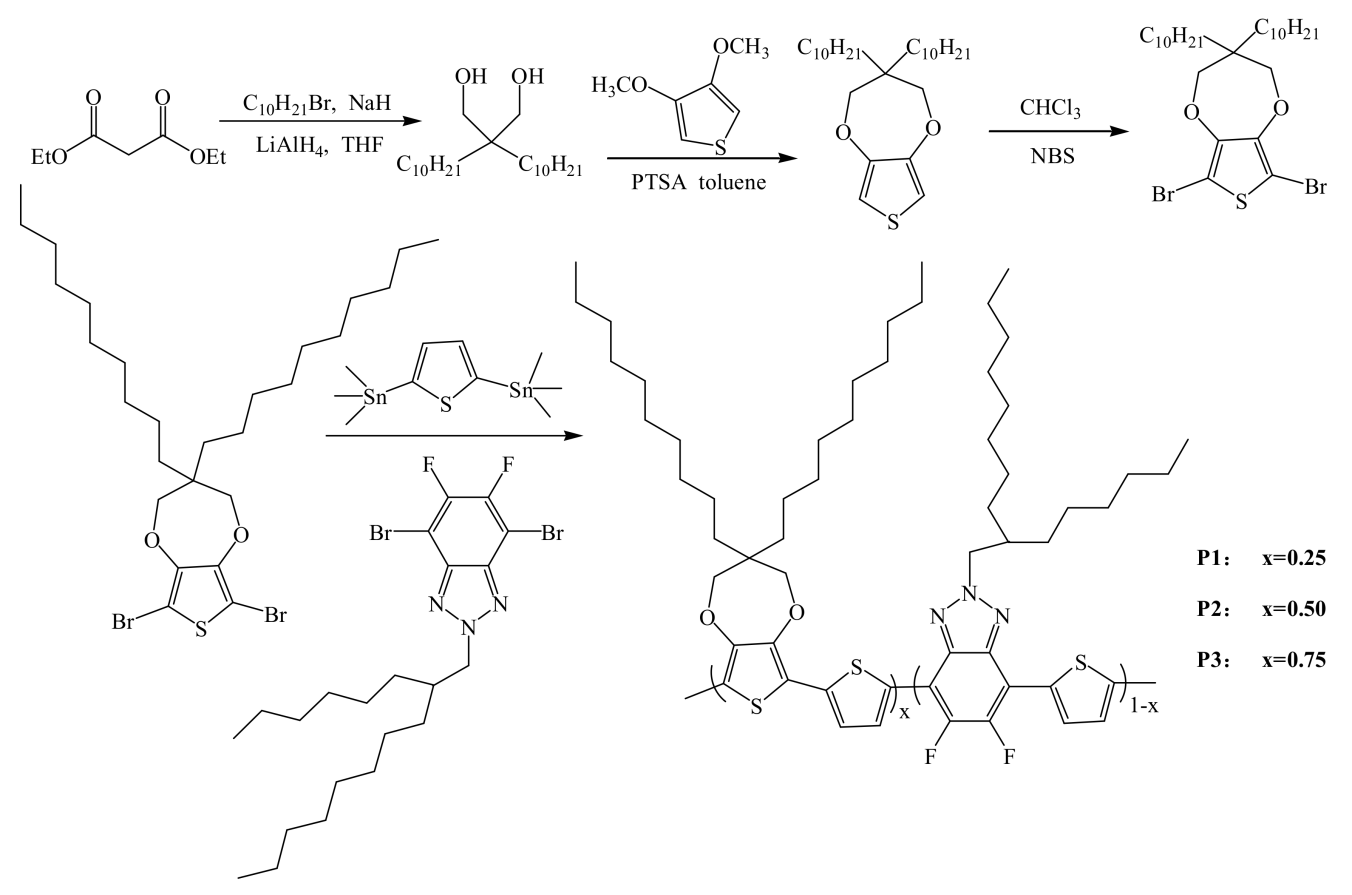
Synthesis steps of monomers and polymer **P1**, **P2** and **P3**.

ProDOT-decyl_2_ was synthesized according to the previous literature [33], and the synthetic route is shown in Scheme 1. 11.5 mmol of diethyl malonate was added dropwise to a mixed solution containing 35 mmol of 1-bromododecane, 35 mmol of sodium hydride, and 200 mL of anhydrous tetrahydrofuran, and then the mixture was stirred under reflux for 24 h under an argon atmosphere. After the completion of the reaction, the mixture was poured into 200 mL of brine and extracted twice with ether. The excess solvent and 1-bromododecane was removed by vacuum distillation and the remaining product was alkylated diethyl malonate (ADM). In the subsequent reaction, the obtained ADM was added dropwise to a solution of 20 mmol of lithium aluminum hydride in 200 mL of diethyl ether. The mixed solution was stirred at room temperature and under an argon atmosphere for 20 h. Several operations were conducted successively, which included filtration, rinsing with ethyl ether, and vacuum distillation (solvent removal). The resultant product was 2,2-didodecylpropane-1,3-diol (DDP). For the preparation of ProDOT-decyl_2_, 20 mmol of the resulting diol (DDP), 15 mmol of 3,4-dimethoxythiophene, and 1.8 mmol of PTSA were added to a single-necked round bottom flask containing 250 mL of toluene. The flask was connected to a Soxhlet extractor with 4A molecular sieves as dehydrating agent. The reaction mixture was stirred and refluxed under argon atmosphere for 24 h. After cooling, the mixture was washed with deionized water and the solvent was removed by vacuum distillation. The resulting crude product was purified by column chromatography on silica gel and eluted with hexane and dichloromethane in a volume ratio of 3:2. Pure ProDOT-decyl_2_ was obtained as yellow oil with 65% yield. ^1^H NMR (CDCl_3_, 400 MHz, δ ppm): 6.42 (s, 2H), 3.84 (s, 4H), 1.26 (m, 36H), 0.88 (t, 6H). ^13^C NMR (CDCl_3_, 101 MHz, ppm): 149.69, 104.63, 77.53, 77.00, 76.68, 43.72, 29.63, 29.33, 22.68, 14.13 (see in Supporting Information (SI), Figure S1).

#### 2.3.2. Synthesis of M1

M1 was also synthesized according to the previous literature [33]. 5 g (11.45 mmol) of ProDOT-decyl_2_ was dissolved in a three-necked round bottom flask with 200 mL mixed solution of chloroform and acetic acid (1:1, *v*/*v*). A solution of 4.89 g (27.47 mmol) of NBS in 100 mL of chloroform was added dropwise with stirring. The reaction was carried out at room temperature for 24 h. Then, the reaction mixture was rinsed with water and extracted with chloroform three times. The organic layer was dried over magnesium sulfate and the solvent was removed by vacuum distillation. The resulting crude product was purified by column chromatography on silica gel, and eluted with a mixture solution of hexane and dichloromethane (*v*/*v*, 5:1) to give the pure, clear oily product (Yield, 88%). ^1^H NMR (CDCl_3_, 400 MHz, δ ppm): 3.90 (s, 4H), 1.26 (m, 36H), 0.88 (t, 6H). ^13^C NMR (CDCl_3_, 101 MHz, ppm): 147.14, 90.63, 77.98, 77.00, 76.68, 43.99, 31.65, 29.72, 22.69, 14.10 (see in SI, Figure S2). 

#### 2.3.3. Synthesis of **P1**, **P2** and **P3**

**P1** polymer was prepared with a feed ratio of 1(M1):3(M2):4(M3). A mixture of 109 mg (0.183 mmol) of M1, 295.1 mg (0.549 mmol) of M2, 300 mg (0.732 mmol) of M3, and 21.78 mg of Pd(PPh_3_)_2_Cl_2_ were added in a round bottom flask containing 50 mL of toluene. The air in the apparatus was evacuated and filled with argon, and this process was repeated four times. The reaction then proceeded at 105 °C for 48 h. After the completion of the reaction, the toluene was removed by distillation under reduced pressure, and the residuum on the inner wall of the flask was scraped off and extracted with methanol and acetone as solvent in turn for 24 h or more until the solution was colorless [34]. The finally resulting polymer **P1** was a maroon solid with a production yield of 85%. ^1^H NMR (400 MHz, CDCl_3_, δ ppm) 8.502–8.056 (m, 3H), 7.211–6.986 (d, 2H), 4.993–4.457 (d, 6H), 4.331–3.727 (s, 4H), 2.076–1.947 (s, 1H), 1.691–1.052 (m, 174H), 0.995–0.687 (m, 33H) (see in SI, Figure S3a). The molecular weight was analyzed by the GPC method and acetonitrile was used as the mobile phase. PMMA was used as the standard. (For **P1**, *M*_n_ = 15.2 k, *M*_w_ = 19.8 k, polymer dispersity index (PDI) = 1.30.) 

**P2** was synthesized in the same way as that of **P1**, and the feed ratio of the monomers was 1(M1):1(M2):2(M3). The mixtures involved in the reaction were 217.7 mg (0.366 mmol) of M1, 196.7 mg (0.366 mmol) of M2, 300 mg (0.732 mmol) of M3, and 23.52 mg of Pd(PPh_3_)_2_Cl_2_, respectively. And, the resulting polymer **P2** was a scarlet solid with a yield of 86%. ^1^H NMR (400 MHz, CDCl_3_, δ ppm) 8.468–8.046 (m, 1H), 7.223–6.893 (d, 4H), 4.899–4.518 (d, 2H), 4.340–3.772 (s, 7H), 2.078–1.942 (s, 1H), 1.734–1.026 (m, 114H), 0.994–0.776 (m, 19H) (see in SI, Figure S3b). The molecular weight test result of **P2**: *M*_n_ = 14.7 k, *M*_w_ = 18.9 k, PDI = 1.29. 

**P3** was also obtained, and the feed ratio of the monomers was 3(M1):4(M2):1(M3) as shown in Scheme 1. The polymer **P3** was obtained as a red solid with a yield of 89%. ^1^H NMR (400 MHz, CDCl_3_, δ ppm) 7.230–6.866 (d, 21H), 4.733–4.592 (d, 1H), 4.330–3.759 (s, 34H), 2.081–1.946 (s, 2H), 1.601–0.996 (m, 617H), 0.972–0.657 (m, 19H) (see in SI, Figure S3c). The molecular weight of **P3**: *M*_n_ = 15.2 k, *M*_w_ = 19.1 k, PDI = 1.26. 

## 3. Results and Discussion

### 3.1. FT-IR Spectra

**Figure 1:**
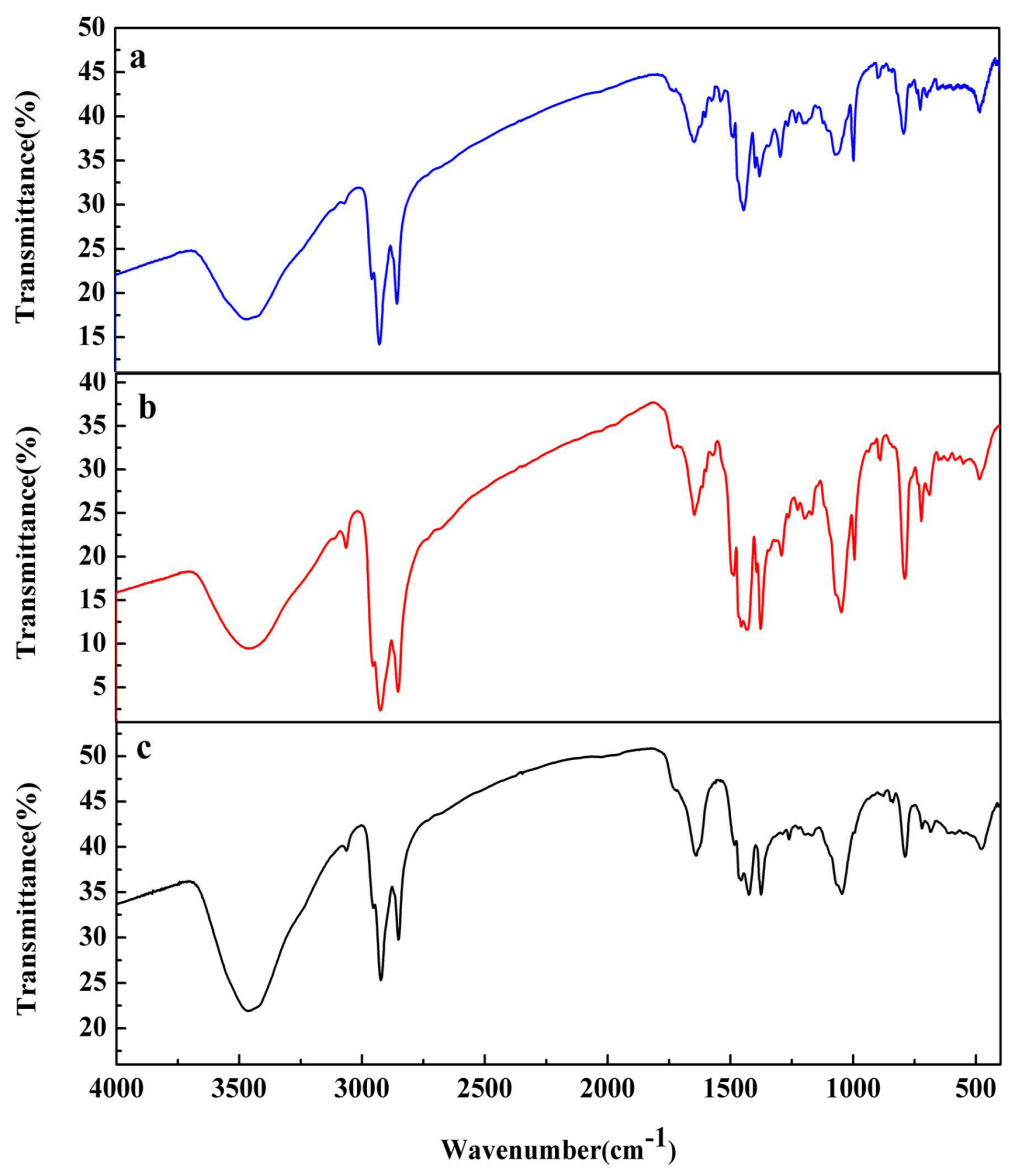
FT-IR spectra of **P1** (**a**); **P2** (**b**); and **P3** (**c**).

The FI-IR spectra of the three polymers were measured to confirm their compositions, and the results are shown in Figure 1. 

As can be seen from the IR spectra of the polymers, the absorption peak locations were nearly the same for all polymers due to the same monomer constitution of the polymers. Except that the intensities of the corresponding peaks of the polymers varied due to the different feed ratios of monomers in the polymers. In terms of **P3**, the absorption peak located at 684 cm^−1^ was due to the bending vibration of two adjacent hydrogen atoms on the M3 unit. The broad peak at 1045 cm^−1^ was due to the stretching vibrations of C–O and C–S bonds in the M1 and M3 units. The absorption peaks at 1374 and 1421 cm^−1^ were due to the bending vibrations of the C–H bonds in methyl and methylene groups of the saturated side chain (M1 or M2), respectively [34]. In addition, the peak at 2851 cm^−1^ was due to the stretching vibrations of the C–H bonds on the tertiary carbon of the alkyl side chain of the M2 unit, and the peak at 2923 cm^−1^ was correlated with the stretching vibrations of the C–H bonds of the methylene unit on the side chains of M1 and M2. Finally, the peak at 3010 cm^−1^ might be due to the stretching vibrations of the C–H bonds on the thiophene ring (M3 unit). The bands at 1424 and 1639 cm^−1^ might be due to the skeleton vibration of the thiophene ring (M1 or M3) and the benzene ring, respectively. From the above analysis, it can be concluded that **P3** consisted of M1, M2, and M3, which confirmed the formation of the copolymer through the coupling reaction. According to the experimental data mentioned above, all of the three copolymers have high molecular weights and low PDI values, which might favor the electrochemical stabilities of the polymers in several aspects, including the improvement of the adhesion to the substrate, the resistant to the dissolve in the supporting electrolyte, and the maintenance of a high optical contrast after repeated electrochromic switches [35].

### 3.2. Electrochemical Characterization

**Figure 2:**
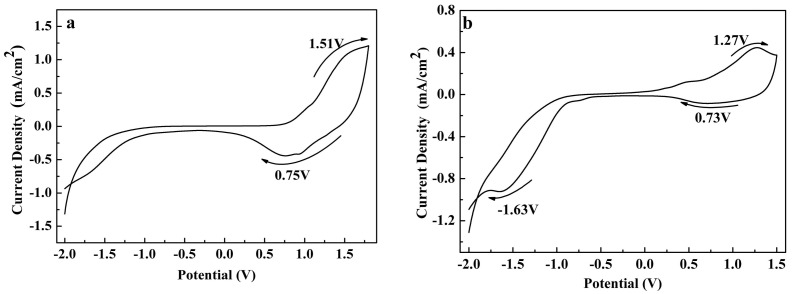

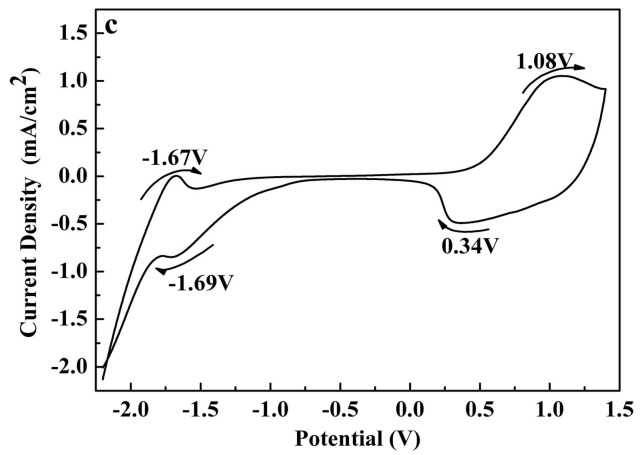
Cyclic voltammograms of the polymer films of **P1** (**a**); **P2** (**b**) and **P3** (**c**).

Spray coated Pt-disk electrodes with the polymers were used as the working electrode to investigate the redox properties of the polymers with the cyclic voltammetry (CV) method. The measurements were conducted in an electrolyte of 0.1 M TBABF_4_/ACN with a potential window between −2.0–1.5 V (vs. pseudo Ag wire reference electrode), and the sweep rate was 100 mV/s. As depicted in Figure 2, all three polymers presented a pair of redox peaks during the p-doping process, and the positions of the redox peaks were located at 1.51 V/0.75 V, 1.27 V/0.73 V, 1.08 V/0.34 V, for **P1**, **P2**, and **P3**, respectively. Under low potentials during the positive scan, the polymers were in a neutral state and carried no charge, and then the polymers were gradually oxidized over the onset oxidation potentials (*E*_onset_) and neutralized by the doping of the anion of the supporting electrolyte. The three polymers had descending *E*_onset_ values from **P1** to **P3**, and the data were 0.89, 0.55 and 0.49 V, respectively (Figure 2). As the oxidation potentials and the degree of oxidation increase, the cationic radicals appeared on the backbone of the polymers, known as polaron, bipolarons etc., which represent the main charge carriers within the conjugated polymers. The three polymers were synthesized with the same monomers, except that the feed ratios between the donor units and acceptor unit were different from each other. It is obvious to find that with the increase of the M1 content in the copolymer, i.e., the increase in the ratio of the total donor units to the acceptor unit (M1 + M3/M2), the redox peak potentials (as well as the *E*_onset_ values) decreased successively from **P1** to **P3**. In addition, of the three copolymers, **P3** had the most well defined redox peaks, which indicated the smoothest doping-dedoping process among the three polymers. The oxidation of **P3** at the lowest oxidation potential was an indication of the highest conjugation length of **P3** among the three polymers, which was caused by the highest content of the M1 unit in **P3**. M1 (ProDOT-decyl_2_) unit possess a characteristic of a strong electronic donor due to the overlap between the lone pairs of electrons, oxygen atom, and thiophene ring, which usually lead to its strong electron donating ability. The HOMO levels of D-A type polymers usually reside on the donor units, and then the increase in the content of the M1 unit would raise the HOMO levels of the polymers, which would finally result in the decrease in *E*_onset_ values [34]. A pair of redox peaks was observed at −1.67 V/−1.69 V (Figure 2c), which indicated that **P3** has the characteristic of n type doping. Only a reduction peak at −1.63 V was observed in the negative potential scan of **P2** (Figure 2b), and not any redox peaks were observed for **P1** in the same process (Figure 2a).

### 3.3. Morphology

**Figure 3:**
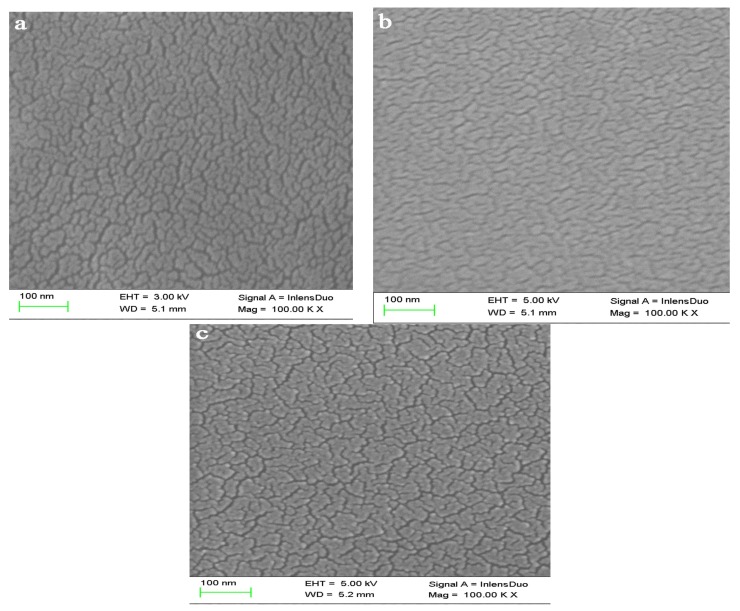
Scanning Electron Microscope (SEM) images of films of **P1** (**a**); **P2** (**b**); and **P3** (**c**).

SEM was used to study the microstructure and bulk morphologies of the polymer films, and the results are presented in Figure 3. According to our previous study, the morphologies of the polymer films had no direct relationships with the structures of the polymer films [34]. The polymer films are not homogeneous from the microcosmic view, which consisted of countless tiny particles separated from each other. The particle sizes of **P1** and **P2** are comparable to each other, and were somewhat smaller than that of **P3**. From the macroscopic view, the polymers have similar texture-like planar structures, which made the polymers “homogenous polymers” from the view of the naked eye. It has been shown that the polymers with smooth and morphological nanostructures often exhibit high performance electrochemical and electrochromic properties [34]. The thicknesses of the polymers (having the same absorption values in the visible light region) were also measured by the step profiler, and the values were 456, 467 and 502 nm, respectively, for **P1**, **P2** and **P3**. 

### 3.4. Optical Properties of Polymer Solutions and Films

**Figure 4:**
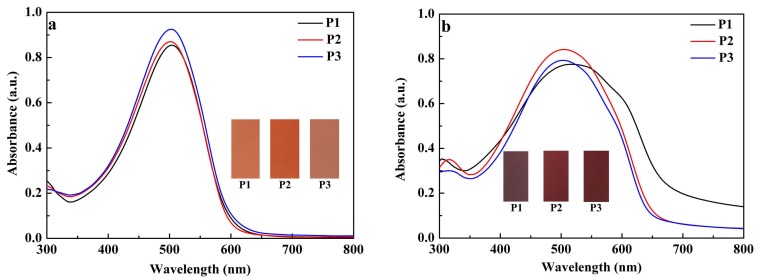
Absorption spectra of three polymers in the form of solutions (**a**) and films (**b**). The inset pictures show the colors of the solutions (**a**) or polymer films (**b**).

The absorption spectra of the three polymers in the form of solutions and films (solid state) were tested by a spectrophotometer, and the results are shown in Figure 4. The polymers were dissolved in chloroform to give solutions of the polymers and then subjected to the test. The polymer films were coated on the ITO electrode with the spray method, and the solution concentration of the polymers for this purpose was 4 mg·mL^−1^. As shown in Figure 4a, the absorption spectra of the three polymer solutions have a well defined and narrow absorption peak in the visible region, and there is no significant difference in the position of the maximum absorption peak, which is 504 nm for **P1**, 502 nm for **P2**, and 502 nm for **P3**, respectively. Therefore, all three polymer solutions exhibit nearly the same orange color. At the same time, the absorption spectra of the polymer films had wider bands compared to their absorption bands in solution, with the maximum peaks at 526, 507 and 502 nm, respectively (Figure 4b). There is a weak attraction force between the molecules of the polymers in the solid state (e.g., polymer film), and the force is called the Van Edward force, which includes orientation force, induction force, and dispersion force. The polymers have a rich content of polar atoms, such as oxygen, sulfur, nitrogen, and fluorine atoms, which are conducive to improving the interaction forces between the segments of the polymers. The diversity of the way of accumulation and the forces between the segments of the polymer chains will inevitably lead to changes in the conjugation and electron absorption of the polymer chains, which might account for the wider electron absorption peaks of the polymers in the solid state (polymer film) than in the solution state. The aggregation and interaction between the molecular chains will increase the conjugation degree of the copolymer to a certain extent, which may be the reason for the redshift of the absorption peak to a certain extent. The relevant data are listed in Table 1 [34].

### 3.5. Spectroelectrochemical Properties

**Figure 5:**
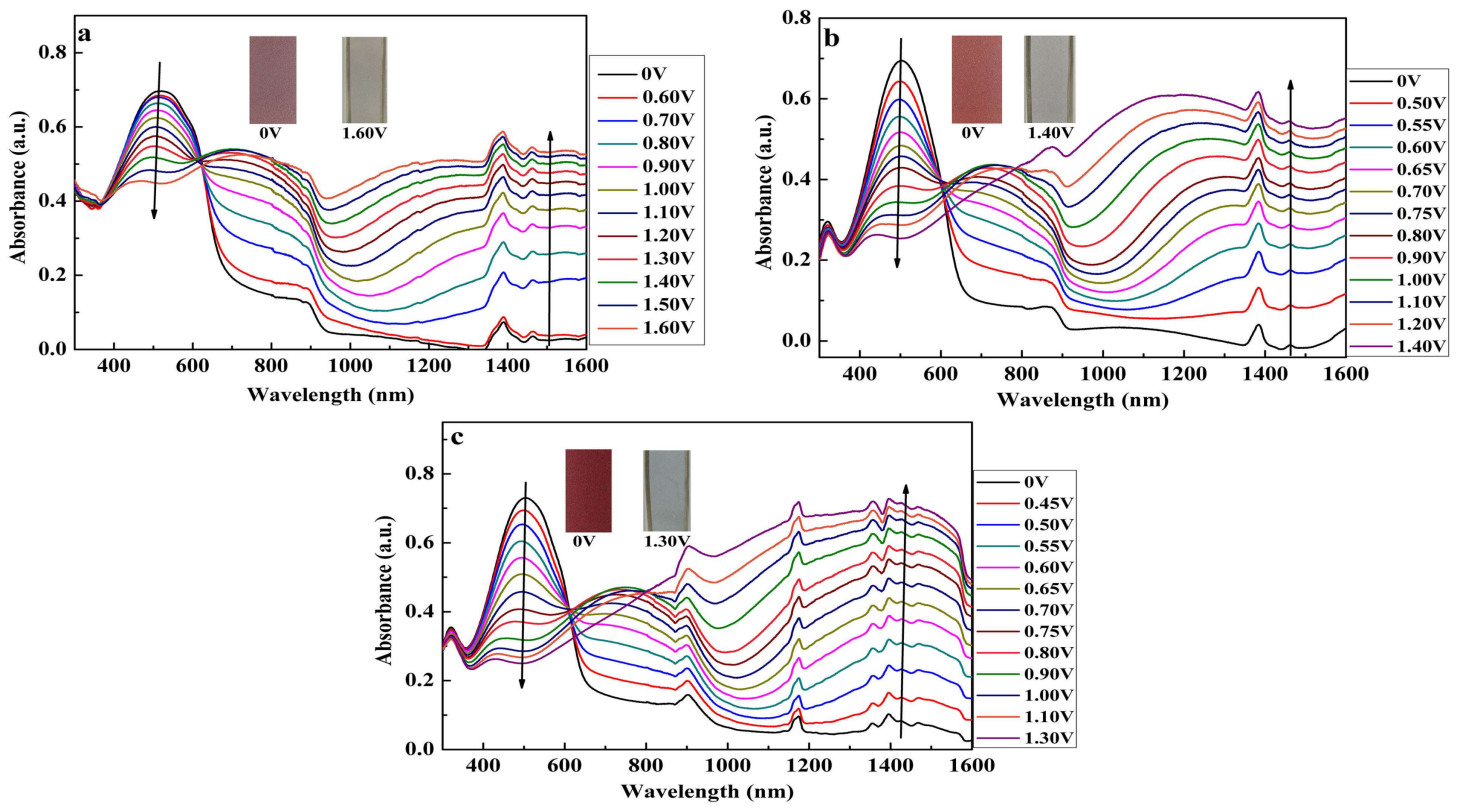
Spectroelectrochemical spectra of **P1** (**a**); **P2** (**b**); and **P3** (**c**) and color change of film under different voltages.

The spectroelectrochemical properties of the polymers were measured by a UV-Vis spectrophotometer connected to an electrochemical workstation. The changes in the electronic absorption along with the increase in the applied voltage was monitored and recorded stepwise from the neutral states to their oxidized states. In the neutral sate, all of the polymers showed a single major absorption peak with the peaks located at 520, 500 and 505 nm, respectively, for **P1**, **P2**, and **P3**, due to the π–π* transition (Figure 5). The π–π* transition band of **P1** is wider than that of **P2** and **P3**, which not only absorbed green and blue light but also absorbed part of the red and yellow lights. As a result, the **P1** polymer presented a reddish brown color, and both **P2** and **P3** exhibited a red color. For **P1**, the electronic absorption began to change at an initial potential of 0.7 V, and then the π–π* transition band decreased continuously, which was accompanied by the occurrence of a newly intensifying band at about 800 nm. The new band was due to the formation of charge carriers (i.e., polarons). Meanwhile, another newly intensifying band was also found beyond 1500 nm, which might be attributed to the formation of bipolarons (Figure 5a). Similar tendencies were also found in the **P2** and **P3** polymer films, and the formation of polarons and bipolarons was also verified by the occurrence of an intensifying band at 730 and 1385 nm for **P2** (Figure 5b), 750 and 1400 nm for **P3** (Figure 5c). **P1** reached a completely oxidized state at a voltage of 1.60 V, and the color switched from a reddish brown color to a light gray color in this process (Figure 5a). The observable absorption change was found for **P2** at 0.5 V (0.45 V for **P3**), and full oxidation was reached at 1.40 V (1.30 V for **P3**). The easier oxidation of **P3** than that of **P1** and **P2** was consistent with the data obtained from the CV tests. For all of three polymers, isosbestic points were observed at 620, 601 and 612 nm, for **P1**, **P2** and **P3**, respectively, suggesting that the polymer films could be interconverted reversibly between the dedoped and doped states. At the fully oxidized states of the polymers, they all exhibited a light gray color, which suggested that three polymers are all cathodically coloring polymers.

The changes in the absorbance of the π–π* transition peaks between the reduced and oxidized states of the polymers were also recorded, and the data were 0.25, 0.44 and 0.48 a.u. respectively, for **P1**, **P2**, and **P3** (Figure 5). Thus, the content of the ProDOT-decyl_2_ unit had a positive relationship with the optical contrasts of the obtained polymers on the π–π* transition peaks. The π–π* transition band of the D-A type polymers usually depends on the donor units, and further, the contrast ratio at this band might also be inherited from the donor units within the polymer to a great extent. As previously reported, the polymer results from the ProDOT moiety functionalized with 2-ethylhexyloxy had an outstanding contrast ratio of 80%. At the same time, the electrochemical oxidation of a conjugated polymer is accompanied by the removal of electrons form the valence band, generating positive charge defects on the backbone, which usually leads to the formation of a quinoidal structure. ProDOT-decyl_2_ not only has the sulfur heteroatom, but also has a strong electron donating ability, which could stabilize the polarons, bipolarons, and the quinones created during the oxidation process. The high content of ProDOT-decyl_2_ unit in **P3** makes the polymer easy to be oxidized, so that **P3** showed the largest contrast ratio among the three polymers due to the largest ProDOT-decyl_2_ content of **P3**. 

The essential parameters of **P1**, **P2**, and **P3** obtained from the electrochemical and spectroelectrochemical test are summarized in Table 1. Where λ_onset_ was determined by the intersection of the absorption peak tangent and the baseline, *E*_g_, *E*_HOMO_ and *E*_LUMO_ were obtained by the formulae *E*_g_ = 1241/λ_onset_, *E*_HOMO_ = −e(*E*_onset_ + 4.4), *E*_LUMO_ = *E*_g_ + *E*_HOMO_, respectively. As shown in Table 1, with the increase in the proportion of donor units, the HOMO values of the three polymers tend to increase, and the bandgap values also increased slightly, but they can still belong to low bandgap polymers [3,4,5]. 

### 3.6. Electrochromic Switching Studies

**Figure 6:**
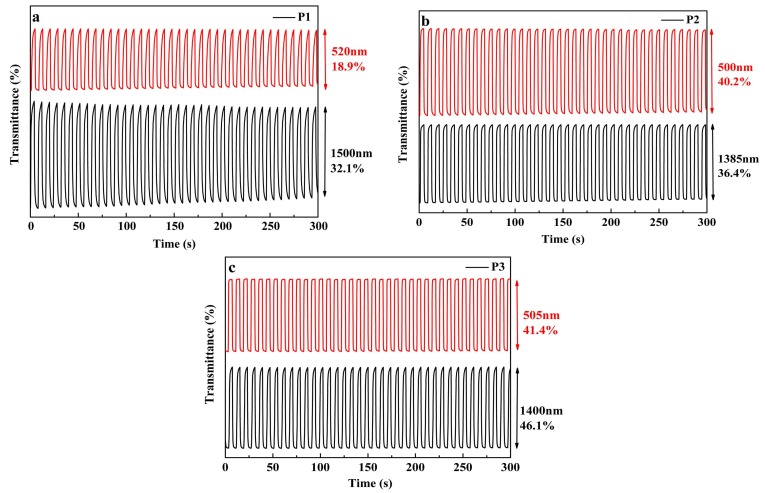
Electrochromic switching of **P1** (**a**); **P2** (**b**); and **P3** (**c**) at different wavelengths.

A kinetic study was conducted for the three polymers to explore their kinetic switching properties, including contrast ratios, response speeds, and stability [36]. For this propose, chronoabsorptometry was used by setting the potentials of the WE electrode (polymer coated ITO electrode) at the reduced potential and the oxidized potential for four seconds, respectively, and the transmittance ratio of the polymers was recorded simultaneously. For polymer **P1**, the contrast ratio (Δ*T*%) values were found to be 18.9% at 520 nm, and 32.1% at 1500 nm (Figure 6a). Response time (*t*_95%_) is usually defined as the time required fulfilling the 95% of the maximum contrast ratio (sensitive to naked eyes) [37]. From the calculation results, oxidation time was measured as 2.56 s at 520 nm, 2.44 s at 1500 nm for **P1**. The switching spectra of **P2** and **P3** were also presented in Figure 6b,c, respectively, and the calculated results are summarized in Table 2. It is apparent that Δ*T*% values increased from **P1** to **P3** in both the visible region and the near infrared region. The Δ*T*% values of **P2** were slightly lower than that of **P3**, with **P3** having a high Δ*T*% value of 41.4% at 505 nm and 46.1% at 1400 nm. As to the *t*_95%_ value, the response time decreased continuously in the visible region from **P1** to **P3**, particularly the 0.54s for **P3** at 505 nm, which is a very fast response time among the polymer based electrochromic materials and can even meet the requirements of non-emissive displays for electrochromic materials [38,39]. However, the same phenomenon was not apparent in the NIR region, as **P3** had a longer response time than that of **P2**. Thus, the high percent ratio of the ProDOT-decyl_2_ unit is helpful to improve the contrast ratios of the resultant conjugated polymers, as well as helpful to the response speeds in general. 

The coloration efficiency (*CE*) was often used to compare the charge as well as the energy utilization efficiency of the related devices [40]. The higher the *CE* value is, the more efficient device could be obtained, which might consume less energy compared with the device with low *CE* values when the same optical contrast is reached [35]. The *CE* value could be obtained by the equations as given below: *CE* = ΔOD/Δ*Q*, ΔOD = log (*T*_b_/*T*_c_), Δ*Q* = *Q*/*A*. *T*_b_ and *T*_c_ refers to the transmittance of polymer in the neutral and oxidized states, respectively, which were obtained from Figure 6. The value of *Q* refers to the charge consumed in a cycle period [34]. A referred to the active area of conductive glass, and was 2.7 cm^2^ in this paper. As shown in Table 2, all three polymers exhibited high *CE* values, especially in the NIR region. 

**Figure 7:**
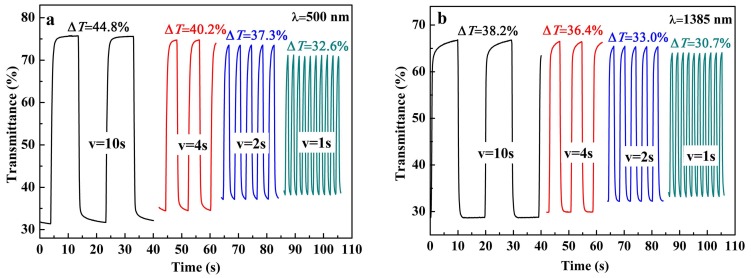
Electrochromic switching of **P2** with intervals of 10, 4, 2 and 1 s, with the detection wavelength at 500 (**a**) and 1385 nm (**b**).

Besides, the changes in Δ*T*% values were also measured by changing the retention times from 10, 4, 2 to 1 s to assay the influence of the retention times on the contrast ratios of the polymers. For **P2**, the Δ*T*% values at 500 nm decreased from 44.8% (10 s), to 40.2% (4 s), 37.3% (2 s), and 32.6% (1 s) (Figure 7a). Meanwhile, the Δ*T*% values were recorded as 38.2%, 36.4%, 33.0%, and 30.7% at the retention times of 10, 4, 2 and 1 s, respectively, at the wavelength of 1385 nm (In the NIR region) (Figure 7b). As the retention times were decreased from 10 to 1 s, the Δ*T*% values were reduced by 26.74% and 19.63% at 500 and 1385 nm, respectively. For **P1**, the retention times had a greater impact on the Δ*T*% values than that of **P2** (Figure S4). Specifically, the reduction rates were 52.96% and 58.1%, respectively, at 520 and 1500 nm, as the retention times changed from 10 to 1 s. Surprisingly, the reduction rate in contrast ratio for **P3** was only 8% as the retention time changed from 10 to 1 s at 505 nm, which suggested the highest stability in optical contrast among the three polymers as the retention times decreased from 10 to 1 s (Figure S5). The contrast ratio of **P3** at 1400 nm was reduced by 35.6% as the retention time changed from 10 to 1 s (Figure S5b), which was more sensitive to the changes in retention times than that of the shorter wavelength. The changes in the transmittance ratios are determined by the doping (oxidation) and dedoping (reduction) of the polymers, which is characterized by the diffuse into or diffuse out of the polymer films by the counter anions. In this case, the sensitivity of the contrast ratios to the retention times might have a relationship with the diffusion capacities of the counter anions in the polymer films. The high diffusion ability of the counter anion within the conjugated polymer might be helpful to keep the contrast ratios, even if the retention times have been changed in a larger range [41,42,43]. As discussed above, the stronger interaction forces between the segments of **P1** might be anticipated rather than that of **P2** and **P3** due to the higher content of M2 units, which will lead to a more compact microstructure of **P1** than that of the two other polymers. And as a result, the diffusion speed of the counter anion in **P1** might be slower than in that of in **P2** and **P3**, which could explain the different sensitivities of the contrast ratios to the retention times of three polymers. 

### 3.7. Colorimetry

**Figure 8:**
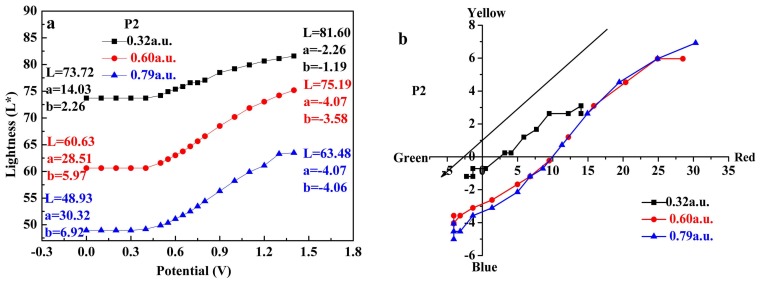
L* and a*, b* curves of **P2** at different applied voltages.

The CIE 1976 L*, a*, b* color space calculated from the transmittance spectra of the **P2** at different biased potentials using air as a background is presented in Figure 8. Films with different thickness were used for the display of the changes in chromaticity. In the neutral state of the **P2**, the L*, a*, b* values increased with the increase of the thickness of the polymers (Figure 8a), which also applied to the **P2** at oxidized states with different thickness. 

The L* values of **P2** gradually decreased as the thickness of the film increased, ranging from 73.72 (0.32 a.u.) to 60.63 (0.60 a.u.) and 48.93 (0.79 a.u.) at a potential of 0 V, which suggested that **P2** shows a gradually deeper color with the increase of thickness. The value of a* represents the red (positive value) and green color (negative value), the larger of the a* value, the more saturated red color of the polymer film is [33]. The polymers at the neutral state with different thickness showed a high a* value and a low b* value (Figure 8a), which indicated that the polymers presented more and more saturated red color as their thickness increased. At the oxidized state, the **P2** polymer film (a.u. 0.32) showed high L* (81.60) and low color opponent dimensions (a* = −2.26, b* = −1.19), indicating high transparency and colorlessness at the oxidized state. The polymer films of **P2** with different thickness also showed a similar feature. The changing trend of color with voltages for **P2** film could be more vividly presented in Figure 8b. The arrow in the figure showed the trend of the color change as the polymer films (**P2**) were gradually oxidized. Films with different thickness showed a consistent color change, i.e., the red color in the neutral state changed to a lilac color at the intermediate voltage, and finally changed to a pale gray color at the full oxidized state. The variation of colors with potential change for **P1** and **P3** was similar with that of **P2**, and the data are presented in Figures S6 and S7, respectively. Polymer **P1** showed a reddish brown color at the neutral state, which was changed to a nearly colorless state at the oxidized state (Figure S6a,b). Accordingly, polymer **P3** showed a reddish color at the neutral state, and then changed to transparent light blue color at the oxidized state (Figure S7a,b). 

### 3.8. Thermogravimetric Analysis

**Figure 9:**
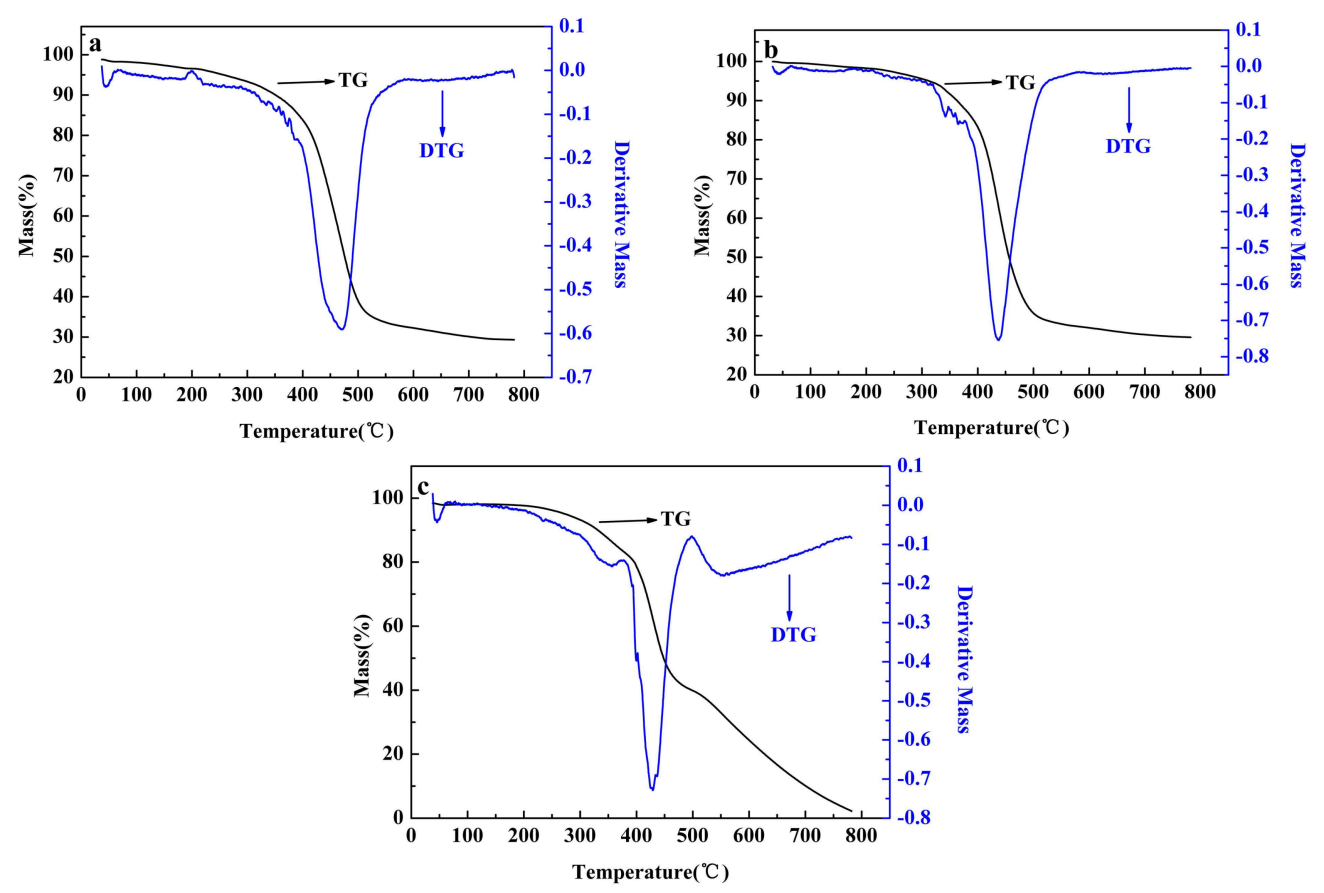
Thermogravimetric analysis of **P1** (**a**); **P2** (**b**); and **P3** (**c**).

Thermal gravimetric (TG) analysis was adopted to study and compare the thermal stabilities of the polymers, based on which differential thermal gravity (DTG) curves were also obtained, and the data were shown in Figure 9 [34]. The initial decomposition temperature (IDT) was obtained from the intersection points between the tangent lines of the baseline and the degradation curves. The IDT data for three polymers were 387, 378 and 373 °C, respectively, with **P1** had the highest IDT data. From the DTG curve, it is convenient to obtain the temperature point, at which the maximum degradation rate was observed, and the data were found at 472, 435 and 428 °C, for **P1**, **P2** and **P3**, respectively. The residual mass percentages of the polymers were also obtained from the TG curve, which were 29.3%, 29.6%, and 2.19%, respectively. Form the above data, it is suggested that all three polymers have perfect temperature tolerance properties, since electrochromic devices usually work at normal temperature conditions. Among the three polymers, **P1** showed the highest thermal stability, and was followed by **P2** and **P3** in turn. The results confirmed that the introduction of the fluorine containing FBTA units had a positive effect on the stability of the resultant polymers, which was consistent with the previous reports on this point [37]. 

## 4. Conclusions

In this work, three random D-A type copolymers were successfully synthesized via a Stille coupling reaction based on the ProDOT-decyl_2_, FBTA and thiophene as the constitutional units. Of the three polymers, the molar ratios of the thiophene contents were the same, **P1** had the highest FBTA content, and **P3** had the highest ProDOT-decyl_2_ content. While the proportion of monomer contents in **P2** was the compromise of **P1** and **P3**. A variety of instrumental analysis methods, including CV, UV-Vis, opto-electronic, chronoabsorptometry and TG-DTG methods, were employed to analyze the relationship between the structure of the polymers and their properties. The *E*_onset_ values of the three polymers exhibited a decremental trend with the increase of proportion of the ProDOT-decyl_2_ unit, indicating that the HOMO levels of the polymers were determined by the donor units of the polymers. The bathochromic-shift of **P1** in the solid state (polymer film) with respect to **P2** and **P3** might arise from the high content of FBTA units, which were assumed to enhance the interactive forces between the polymer segments as well as the formation of the compact microstructures of the polymers. The increase in the ProDOT-decyl_2_ contents is beneficial to the electrochromic properties of the resultant polymers, including contrast ratios and response speeds. Finally, the TG-DTG measurement confirmed that the introduction of fluorine-containing FBTA unit is beneficial to the improvement of the thermal stability of the resultant polymers. 

## Figures and Tables

**Table 1:** Summary of essential parameters of **P1**, **P2**, and **P3**.

Polymer	λ_onset_ (film) (nm)	λ_max_ (solution) (nm)	λ_max_ (film) (nm)	*E*_onset_ (V)	*E*_g_ (eV)	HOMO (eV)	LUMO (eV)
**P1**	703	504	526	0.89	1.77	−5.29	−3.52
**P2**	674	502	507	0.55	1.84	−4.95	−3.11
**P3**	668	502	502	0.49	1.86	−4.89	−3.03

**Table 2:** Optical contrast (Δ*T*%), response time (*t*_95%_) and coloration efficiency (*CE*) of three polymers.

Polymer	λ (nm)	Δ*T*% (%)	Response Time (*t*_95%_) (s)	*CE* Value (cm^2^·C^−1^)
**P1**	520 1500	18.9 32.1	2.56 2.44	105.54 178.22
**P2**	500 1385	40.2 36.4	1.31 1.03	170.76 184.40
**P3**	505 1400	41.4 46.1	0.54 2.00	110.62 364.95

## Supplementary Materials

The following are available online at http://www.mdpi.com/2073-4360/10/4/427/s1, Figure S1: ^1^H NMR spectrum of 3,3-didecyl-3,4-dihydro-2*H*-thieno[3,4-*b*][1,4] dioxepine (**a**), CDCl_3_ Solvent peak and water speak were marked by “x”, “y” respectively, ^13^C NMR spectrum of 3,3-didecyl-3,4-dihydro-2*H*-thieno[3,4-*b*][1,4] dioxepine (**b**), CDCl_3_ Solvent peak were marked by “x”. Figure S2: ^1^H NMR spectrum of 6,8-dibromo-3,3-didecyl-3,4-dihydro-2*H*-thieno [3,4-*b*][1,4]dioxepine (**a**), CDCl_3_ Solvent peak and water speak were marked by “x”, “y” respectively, ^13^C NMR spectrum of 6,8-dibromo-3,3-didecyl-3,4-dihydro-2*H*-thieno[3,4-*b*][1,4]dioxepine (**b**), CHCl_3_ Solvent peak were marked by “x”. Figure S3: ^1^H NMR spectrum of **P1**(**a**), **P2**(**b**), **P3**(**c**), CDCl_3_ Solvent and tetramethylsilane peaks were marked by “x”, “y” respectively. Figure S4: Electrochromic switching of **P1** with intervals of 10, 4, 2 and 1 s. Figure S5: Electrochromic switching of **P3** with intervals of 1, 4, 2 and 1 s. Figure S6: *L** and *a**, *b** curves of **P1** at different applied voltages. Figure S7: *L** and *a**, *b** curves of **P3** at different applied voltages.
