# Supplementary material for: Donor-Acceptor-Type Copolymers Based on 3,4-Propylenedioxy-thiophene and 5,6-Difluorobenzotriazole: Synthesis and Electrochromic Properties
Source: Polymers (Basel). 2018 Apr 11;10(4):427. doi: 10.3390/polym10040427 (PMC6415233; doi:10.3390/polym10040427)
Supplement: Supplementary file 1 [file polymers-10-00427-s001.pdf]

Supplementary Electronic Materials

Donor-acceptor-type Copolymers Based on 3,4-Propylenedioxy-thiophene and 5,6-Difluorobenzotriazole: Synthesis and Electrochromic Properties

Fanda Feng[1], Lingqian Kong[2], Hongmei Du[1], Jinsheng Zhao[1,*], Junhong Zhang[1,*]

[1]Shandong Key Laboratory of Chemical Energy Storage and Novel Cell Technology, Liaocheng University, Liaocheng, 252059, China; 18863500270@163.com (F.F.); duhongmei@lcu.edu.cn (H.D.)

[2]Dongchang College, Liaocheng University, Liaocheng, 252059, China; lingqiankong@126.com

Corresponding author: zhaojinsheng@lcu.edu.cn (J.Zhao); zhangjunhong@lcu.edu.cn (J.Zhang).

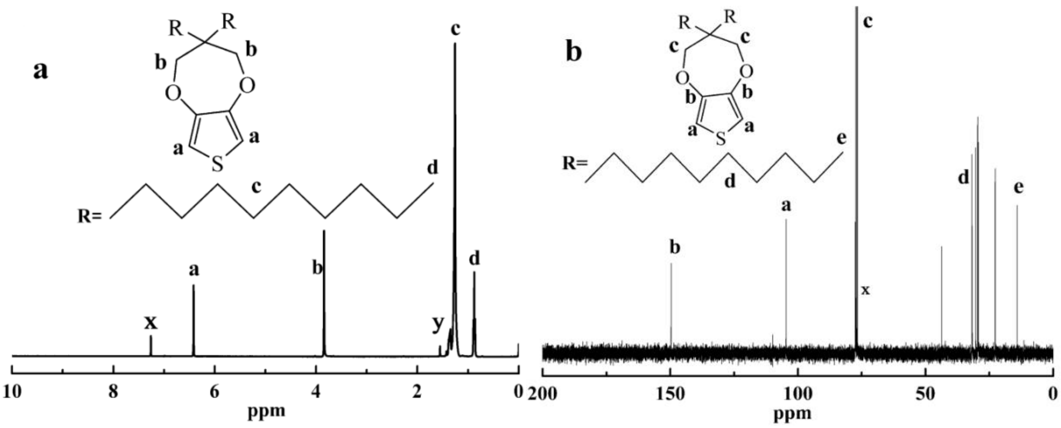

Figure S1. ^{1}H NMR spectrum of 3,3-didecyl-3,4-dihydro-2H-thieno[3,4-b][1,4] dioxepine (**a**), $CDCl_3$ Solvent peak and water speak were marked by “x”, “y” respectively, ^{13}C NMR spectrum of 3,3-didecyl-3,4-dihydro-2H-thieno[3,4-b][1,4] dioxepine (**b**), $CDCl_3$ Solvent peak were marked by “x”.

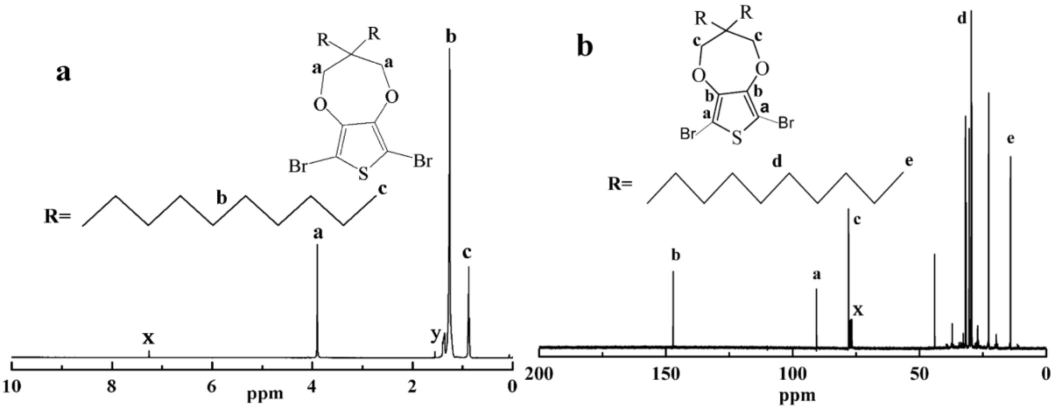

Figure S2. ^{1}H NMR spectrum of 6,8-dibromo-3,3-didecyl-3,4-dihydro-2H-thieno [3,4-b][1,4]dioxepine (**a**), $CDCl_3$ Solvent peak and water speak were marked by “x”, “y” respectively, ^{13}C NMR spectrum of 6,8-dibromo-3,3-didecyl-3,4-dihydro-2H-thieno[3,4-b][1,4]dioxepine (**b**), $CHCl_3$ Solvent peak were marked by “x”.

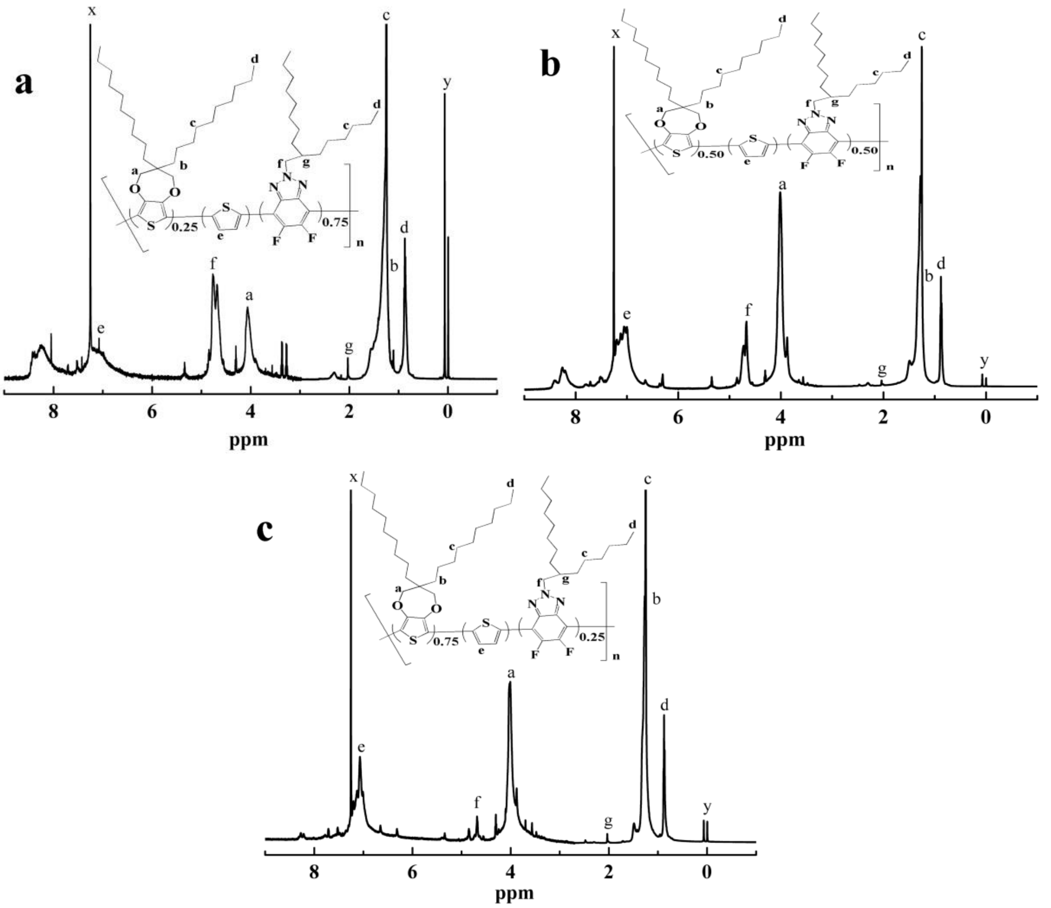

Figure S3. ^{1}H NMR spectrum of **P1**(**a**), **P2**(**b**), **P3**(**c**), $CDCl_3$ Solvent and tetramethylsilane peaks were marked by “x”, “y” respectively.

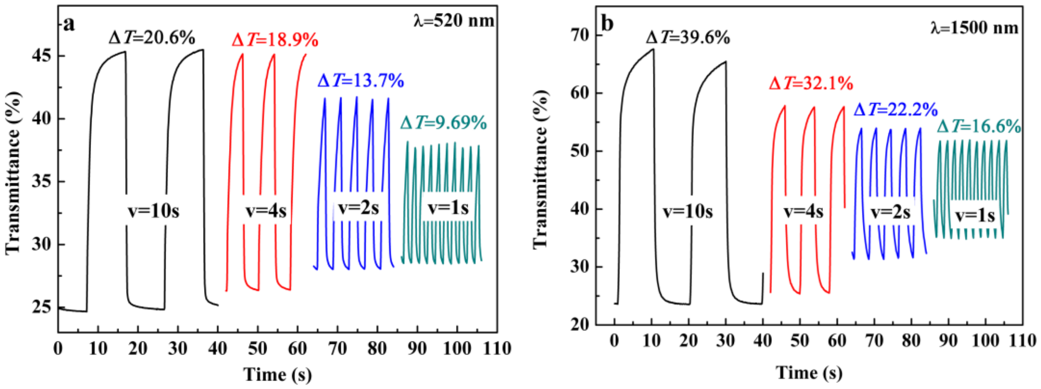

Figure S4. Electrochromic switching of **P1** with intervals of 10, 4, 2 and 1 s.

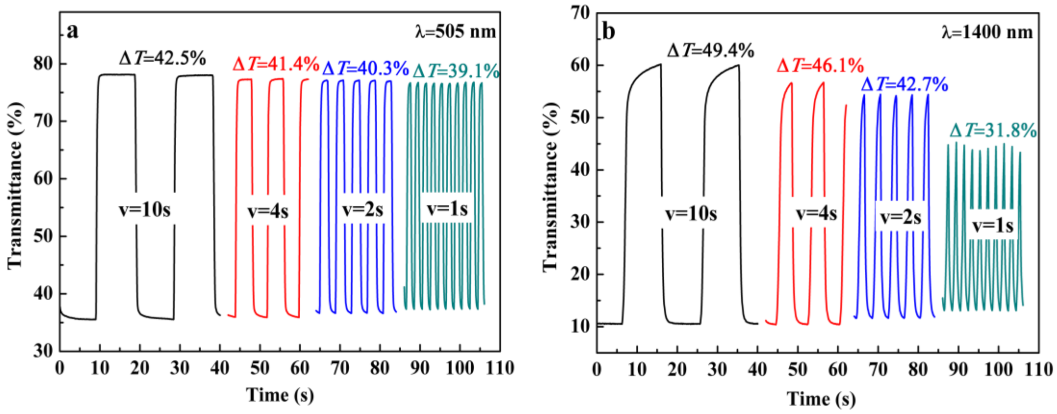

Figure S5. Electrochromic switching of **P3** with intervals of 1, 4, 2 and 1 s.

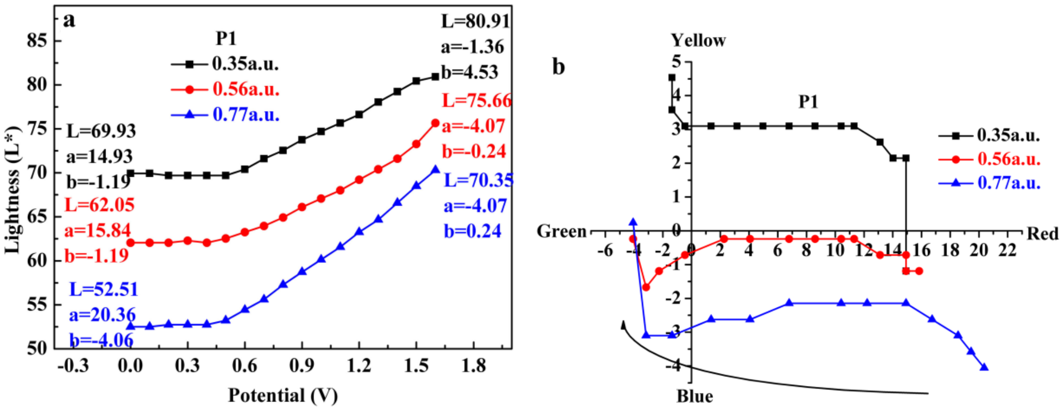

Figure S6. *L** and *a**, *b** curves of **P1** at different applied voltages.

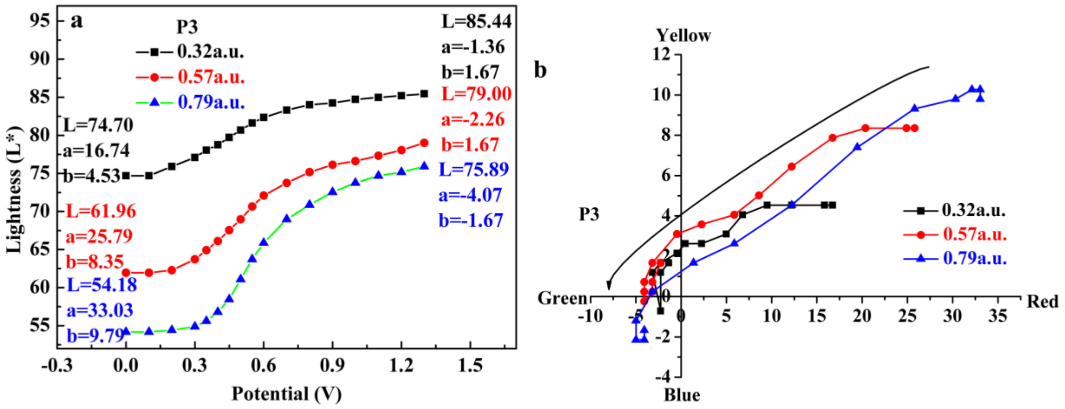

Figure S7. L^* and a^*, b^* curves of **P3** at different applied voltages.
